# A Quantitative Study of the Effects of Guest Flexibility on Binding Inside a Coordination Cage Host

**DOI:** 10.1002/chem.201604796

**Published:** 2016-11-23

**Authors:** Christopher G. P. Taylor, William Cullen, Olivia M. Collier, Michael D. Ward

**Affiliations:** ^1^ Department of Chemistry University of Sheffield Sheffield S3 7HF UK

**Keywords:** binding constants, coordination cage, host–guest chemistry, self-assembly, virtual screen

## Abstract

We have performed a systematic investigation of the effects of guest flexibility on their ability to bind in the cavity of a coordination cage host in water, using two sets of isomeric aliphatic ketones that differ only in the branching patterns of their alkyl chains. Apart from the expected increase in binding strength for C_9_ over C_7_ ketones associated with their greater hydrophobic surface area, within each isomeric set there is a clear inverse correlation between binding free energy and guest flexibility, associated with loss of conformational entropy. This can be parameterized by the number of rotatable C−C bonds in the guest, with each additional rotatable bond resulting in a penalty of around 2 kJ mol^−1^ in the binding free energy, in good agreement with values obtained from protein/ligand binding studies. We used the binding data for the new flexible guests to improve the scoring function that we had previously developed that allowed us to predict binding constants of relatively rigid guests in the cage cavity using the molecular docking programme GOLD (Genetic Optimisation of Ligand Docking). This improved scoring function resulted in a significant improvement in the ability of GOLD to predict binding constants for flexible guests, without any detriment to its ability to predict binding for more rigid guests.

## Introduction

Self‐assembled coordination cages are of current intense interest because they provide a way to prepare hollow containers with a wide range of shapes and sizes that have the ability to bind guest molecules in their central cavities.^1^, ^2^, ^3^, ^4^, ^5^, ^6^, ^7^ This can lead to a wide range of useful types of behaviour, including stabilisation of guest molecules that would otherwise be reactive;^2^ sensing of bound guests;^3^ catalytic transformations of bound guests;^4^ and controlled uptake/ release of drug molecules.^5^

All of this requires a good understanding of which guests will bind in which cage cavities. In addition to obvious issues such as shape and size matching of the guest with the cavity of the host, more subtle factors affecting guest binding include electronic interactions between the guest and the cavity internal surface, desolvation of both guest and cavity surface when the guest binds, and conformational changes associated with folding flexible guests to fit in confined spaces. The majority of reports on guest binding in synthetic containers have relied on empirical approaches to identifying appropriate guests, with a substantial trial and error component: in contrast more systematic approaches, that attempt to quantify the various contributions to guest binding to allow a degree of prediction in identifying new guests, are very limited.^6^, ^7^

**Figure 1:**
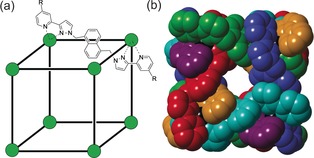
The host cages [Co_8_L_12_](BF_4_)_16_, abbreviated as **H** (R=H, used for crystallographic studies in this paper) and **H^w^** (R=CH_2_OH, used to measure binding constants of guests in water). a) A sketch emphasising the cubic array of Cd^II^ ions and the disposition of a bridging ligand; b) a view of the complex cation of **H** from a crystal structure with each ligand coloured separately for clarity, emphasising the entwined ligand set and the windows in the centre of each face which allow guest entry/ exit.

We recently reported a virtual screening method for identifying new guests for the cavity of our [Co_8_L_12_](BF_4_)_16_ octanuclear cubic coordination cage **H^w^** in water (see Figure 1 for structure; ‘**H**′ denotes the parent organic‐soluble host cage, and **H^w^** denotes the isostructural analogue with hydroxymethyl groups on the exterior surface at the pyridyl C^4^ positions to provide water solubility).^7^ This screening was based on the use of the molecular docking program GOLD (Genetic Optimisation of Ligand Docking), which was developed to model protein/small molecule interactions).^8^ We started with an initial training set of binding constants for >50 guests that had been accumulated empirically, using trial‐and‐error to identify the first guests, followed by extension to other possible guests of comparable size and shape. With this set of data we developed a scoring function that allowed GOLD to predict binding constants for the set of guests that matched the observed binding constants as closely as possible. We then used this scoring function during a screen of a virtual library of untested compounds to identify new potential guests, and from this were able to identify 15 new guests, the predicted and measured binding constants of which were in excellent agreement over a wide range of binding strengths. This single computational screen allowed us, in one calculation taking a few days on a standard PC, to identify several new guests for **H^w^** that were more strongly binding than the best we had been able to find by trial and error during the previous two years.^7^

During this work it became apparent that the flexibility of the guest was a key parameter in its binding strength, with relatively rigid cyclic aliphatic ketones binding strongly but their open‐chain analogues showing very weak or no detectable binding due to the enthalpy and entropy penalty associated with folding up to fit in the host cavity.6c Notably, we found that a particularly strongly binding guest, cycloundecanone, has a conformation when bound (from X‐ray crystallography) that is essentially identical to the calculated minimum‐energy conformation in the gas‐phase; that is, it is almost perfectly preorganised.6c All of the strongly binding guests that we found during our initial empirical studies,3a, 5e, 6c, 9 as well as the additional examples identified from virtual screening,^7^ are relatively rigid and contain at least one ring system. Similarly, during our recent work on binding alkylphosphonate guests,3a we found that GOLD did a notably poor job of predicting their binding strengths; and this got worse as the alkyl chain size increased.

We were therefore interested to probe the effects of guest flexibility further, with a view to i) understanding the extent to which rigidity/flexibility of a series of related guests affects their binding strength; ii) quantifying the enthalpy and entropy effects involved; and iii) improving the scoring function used by GOLD so that its predictive ability could accurately predict binding of flexible guests as well as rigid ones, thereby extending the utility of our in silico screening method for a synthetic host.^7^

## Results and Discussion

### Choice of guests

**Scheme 1:**
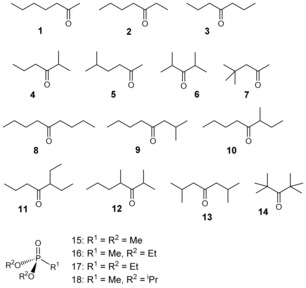
Set of guest molecules evaluated in this work.

To evaluate the effects of guest flexibility on binding we used principally two series of aliphatic ketones: a set of heptanone isomers, and a set of nonanone isomers (Scheme 1). These were chosen for several reasons. Firstly, we know from previous work that cyclic aliphatic ketones can bind in the cage cavity strongly in water, with the high hydrophobic surface area provided by the aliphatic skeleton being the major contribution to binding of these guests.6c Secondly, the polar carbonyl group of the ketone provides sufficient water solubility to enable binding constants to be measured, and also provides an anchoring point for H‐bonding to the internal surface of the cage^10^ that facilitates crystallographic characterisation of cage/guest complexes.3a, 5e, 6c Thirdly, these C_7_ and C_9_ ketones are readily available as a number of isomers with different branching of the alkyl chains, which provides precisely the structural variation that we need in a series of related guests with similar molecular volumes, electronic properties, and hydrophobic surface areas. In addition to the ketones, we included in the guest set a series of four alkyl phosphonates with varying alkyl group sizes, whose binding inside **H^w^** was reported recently.3a

Details of the guests and some of their metric properties (surface area and volume) are summarised in Scheme 1 and Table 1. We note that all of these guests have volumes significantly below 55 % of the cavity volume of **H^w^** (ca. 400 Å^3^) which, as Rebek showed6d, 11 and we have confirmed,6c is approximately the point at which steric bulk starts to inhibit guest binding. Also included in this table is the calculation of the “number of rotatable bonds” (NRB) for each guest; this is the number of bonds which allow free rotation around themselves, and is a term we introduced into the scoring function to take account of the conformational entropy of each guest.^7^ Each C−C single bond that is not in a ring and that has a non‐terminal heavy atom at each end adds 1 to this score, and the parameter NRB has been used in many other calculations of guest binding as a way to take account of the entropic cost of binding arising from loss of flexibility.^12^

**Table 1:** Structural data, and binding data (water, 298 K) for the guests investigated.

Guest	NRB^[a]^	Surface area [Å^2^]	Volume [Å^3^]	*K* [M^−1^]	−Δ*G*° [kJ mol^−1^]
**1**	4	174.5	146.0	59(36)^*b*^	10.1(13)^*b*^
**2**	4	174.2	145.9	72(37)	10.6(14)
**3**	4	174.5	146.0	210(160)	13.2(17)
**4**	3	173.4	145.8	275(160)	13.9(16)
**5**	3	174.5	146.4	204(93)	13.2(12)
**6**	2	172.8	145.5	625(300)	16.0(12)
**7**	2	169.7	145.6	699(300)	16.2(10)
**8**	6	213.8	182.5	863(400)	16.7(10)
**9**	5	215.0	183.2	2040(670)	18.9(9)
**10**	5	212.8	183.0	1590(540)	18.3(9)
**11**	5	213.4	183.2	2620(860)	19.5(9)
**12**	4	207.6	182.8	2440(730)	19.3(8)
**13**	4	210.2	182.3	3890(67)	20.5(1)
**14**	2	199.4	180.8	12100(2000)	23.3(5)
**15**	2	151.0	118.6	7(2)	4.8(6)
**16**	4	192.4	155.5	26(23)	8.1(17)
**17**	5	212.2	174.0	160(45)	12.6(7)
**18**	4	230.2	192.1	390(15)	14.8(1)

[a] NRB=number of rotatable bonds: see main text. [b] Each titration was repeated at least three times, and the experimental error is quoted as twice the standard deviation.

### Structures of cage/guest complexes

We structurally characterised cage/guest complexes with some of the heptanone isomers as guests; we could not obtain good‐quality crystals with any of the nonanone guests. Crystallographic parameters are summarised in Table 2. In both cases the crystalline complexes were prepared by treatment of pre‐formed single crystals of **H** with a concentrated solution of the guest in MeOH, resulting in uptake of guest molecules into the cage cavity without loss of crystallinity. We used **H** rather than **H^w^** for the structural studies as the cage/guest complexes are easier to prepare, and the crystals give better diffraction, than complexes based on **H^w^**. The two cages are isostructural apart from the hydroxymethyl substituents on the exterior surface of **H^w^**, so we can confidently use the structures of complexes of **H** to understand guest binding in the cavity of **H^w^**.

**Table 2:** Crystal parameters, data collection and refinement details for the structures in this paper.

Complex	**H⋅6⋅MeOH**	**H⋅3⋅MeOH**
formula	C_344_H_282_B_16_Co_8_F_64_N_72_O_2_	C_344_H_282_B_16_Co_8_F_64_N_72_O_2_
molecular weight	7316.8	7316.8
*T* [K]	100(2)	100(2)
radiation wavelength [Å]	1.54178	0.71073
crystal system	monoclinic	monoclinic
space group	*C*2*/c*	*C*2*/c*
*V* [Å]	32.9008(15)	32.9184(17)
*V* [Å]	30.1251(13)	29.9311(13)
*V* [Å]	39.7960(16)	39.817(2)
*β* [*°*]	96.327(2)	96.111(3)
*V* [Å^3^]	39203(3)	39008(3)
*Z*	4	4
*ρ* [g cm^−3^]	1.240	1.246
crystal size [mm^3^]	0.15×0.15×0.12	0.2×0.2×0.2
*μ* [mm^−1^]	3.346 (Cu‐Kα)	0.421 (Mo‐Kα)
data, restraints, parameters	34484, 1442, 1818	44822, 2409, 1954
final *R*1, *wR*2^[a]^	0.113, 0.305	0.123, 0.402

[a] The value of *R*1 is based on ‘observed“ data with *I*>2*σ*(*I*); the value of w*R*2 is based on all data.

**Figure 2:**
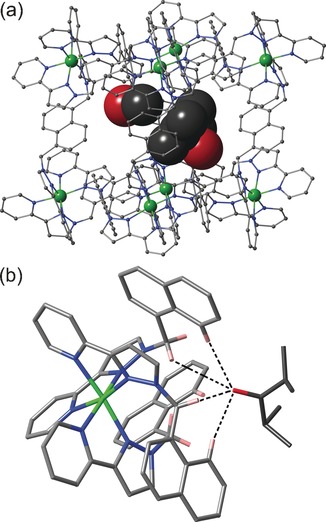
Crystal structure of the supramolecular complex cation of **H⋅6⋅MeOH**: a) a view showing the cage in wireframe with the guests space‐filling; b) a view of one of the *fac* tris‐chelate vertices of the cage showing the H‐bonding interactions with the guest (dashed lines indicate CH⋅⋅⋅O interactions of <3 Å).

**Figure 3:**
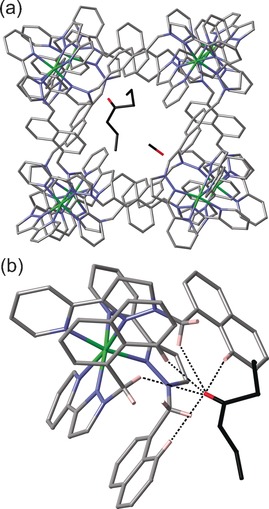
Crystal structure of the supramolecular complex cation of **H⋅3⋅MeOH**: a) a view showing the complete cage and both guests in wireframe; b) a view of one of the *fac* tris‐chelate vertices of the cage showing the H‐bonding interactions with the guest (dashed lines indicate CH⋅⋅⋅O interactions of <3 Å).

In each case it is clear that the guests are oriented inside the cavity via the interaction of the electron‐rich carbonyl oxygen atom with a convergent set of C−H protons from the cage interior surface at a region of high positive electrostatic potential around the two *fac* tris‐chelate vertices.^10^ This provides a set of charge‐assisted CH⋅⋅⋅O interactions, as we have seen with a range of other bound guests in structurally characterised complexes with **H**.3a, 5e, 6c Perhaps unsurprisingly the best‐quality structures with the most crystallographically well‐behaved guests are those when the guest is highly substituted and relatively rigid, or symmetrical; with more flexible and low‐symmetry guests, severe disorder of the alkyl chains was apparent. Figure 2 a shows the structure of the complex **H⋅6⋅MeOH** in which one molecule of 2,4‐dimethyl‐3‐pentanone (**6**) and one of MeOH are located in the two interior binding pockets of the cage, at either end of a long diagonal of the cube (two molecules of **6** would together be substantially too large for the cavity volume based on the Rebek 55 % rule).9a, 10 The presence of a convergent set of CH protons, from the methylene groups and from the naphthyl groups, which form an H‐bond donor pocket around the ketone O atom is clear and several of the CH⋅⋅⋅O distances are less than 3 Å (Figure 2 b). The dipositive charge of the nearby cobalt(II) ion (O⋅⋅⋅Co, 5.61 Å) will assist this interaction.^10^ The structure of complex **H⋅3⋅MeOH,** with one molecule of 4‐heptanone (**3**) and one of MeOH in the cavity, is generally very similar and shown in Figure 3; the non‐bonded O⋅⋅⋅Co distance involving the 4‐heptanone is 5.63 Å.

### Solution binding properties of the guests

Binding constants for all guests were measured in D_2_O by conventional ^1^H NMR titrations; the results are included in Table 1. From the data for the two new guest sets (C_7_ and C_9_ ketones) in this work we can immediately see some striking effects.

Firstly, the C_9_ ketones bind generally more strongly than the C_7_ ketones, which can be simply understood in terms of the greater quantity of hydrophobic material in the former (two additional CH_2_ groups). The strength of hydrophobic binding is related to the combined hydrophobic surface area of host and guest that are desolvated when they come into contact.6c, 9a, 13 In our previous work on binding of the homologous series of cyclic aliphatic ketones, we found that each additional CH_2_ group in the guest afforded an additional 4–5 kJ mol^−1^ of binding free energy when binding in the cavity of **H^w^** in water, with the binding constants for cycloheptanone and cyclononanone being 4.2(4)*x*10^2^ m ^−1^ and 1.1(3)*x*10^4^ m ^−1^ respectively, affording binding free energies (−Δ*G*°) of 15 kJ mol^−1^ and 23 kJ mol^−1^ respectively.6c We can clearly see a similar general difference between the open‐chain C_7_ and C_9_ ketones, although there is spread of values within each group associated with the different degrees of conformational flexibility of the various guests. If however we choose as a matched C_7_/C_9_ pair 2,4‐dimethyl‐pentan‐3‐one (**6**) and 1,1,1,3,3,3‐hexamethylacetone (**14**), both of which have twofold symmetry and NRB=2, we find that their binding parameters (*K=*6.3×10^2^ and 1.2×10^4^ m ^−1^, respectively; −Δ*G*°=16 and 23 kJ mol^−1^, respectively) match very well what was found for their cyclic analogues.6c

Secondly, there is a clear inverse correlation of binding strength with guest flexibility, using the simple NRB parameter as a measure of flexibility. This is apparent in Table 1 in which the guests in each of the C_7_ and C_9_ subgroups are listed in order of their NRB value. For example, within the C_7_ ketone series, it is clear that the two most highly substituted and therefore rigid guests (with NRB=2) are the strongest binders whereas the open‐chain ketones with NRB=4 are the weakest; a similar trend is clear within the C_9_ ketone series. Some simple trends from the available data have been extracted and are summarised in Table 3. Within each family of isomeric guests (C_7_ or C_9_) the values of −Δ*G*° for those guests with a given NRB value have been averaged: for example binding free energy for guests **1** and **2** (C_7_ chain; NRB=4) is 16.1 kJ mol^−1^, which is the first entry in Table 3. Within the C_7_ guest set we can see stepwise reductions in the value of −Δ*G*° of 2.6 and then 2.2 kJ mol^−1^ associated with each stepwise increase in flexibility (increase of 1 in the NRB parameter). Similar behaviour occurs within the C_9_ guest set with stepwise reductions in the value of −Δ*G*° of between 1.3 and 1.7 kJ mol^−1^ per unit increase in the NRB parameter. There is a reasonable degree of consistency across these increment values; given the experimental uncertainty associated with determination of individual −Δ*G*° values we can say that each additional rotatable bond in a guest reduces its binding free energy by about 2 kJ mol^−1^. This compares very favourably with a penalty in the range around 1.5–4 kJ mol^−1^ per rotatable bond that has been calculated for binding of many different flexible substrates to proteins.12b,12c,12g Interestingly, it follows that adding one additional CH_2_ group to a flexible chain, which would create two additional rotatable C−C bonds, will increase the entropic penalty of binding by around 4 kJ mol^−1^, which is comparable to the benefit arising from the increased hydrophobicity of one additional CH_2_ group.6c This agrees with the generalisation made in a recent review that the loss of configurational entropy on binding substrates to proteins can be nearly as large as the gain in favourable binding energy and the two factors tend to correlate.12g

**Table 3:** Average binding free energies of isomers with the same ‘number of rotatable bonds’, with the step changes between them.

Number of C atoms	NRB	−Δ*G*° (average)/ [kJ mol^−1^]	−Δ*G*° increment per NRB [kJ mol^−1^]
7	2	16.1	–
7	3	13.5	2.6
7	4	11.3	2.2
9	2	23.3	–
9	4	19.9	1.7
9	5	18.6	1.3

Finally, we note that within each set of unbranched ketones, the most symmetrical one (4‐heptanone, 5‐nonanone) is significantly more strongly binding than the others despite having the same NRB value, hydrophobic surface area, and so on. For the C_7_ ketones the effect is small but significant (Table 1); within the set of unbranched C_9_ ketones only 5‐nonanone gave a measurable binding constant at all. We ascribe this to simple steric effects. The guest is anchored in the cavity by H‐bonding to a pocket on the interior surface of the cage (see crystal structures above).^10^ For the twofold‐symmetric unbranched ketones the two alkyl chains on either side of the carbonyl group are the same length, which is as short as possible for that series of open‐chain ketones. For the asymmetrically substituted ketones, one chain is necessarily longer than the other which may create steric problems that become more severe for the larger and more asymmetric ketones. With 5‐nonanone there are two C_4_ chains either side of the carbonyl anchoring point, but with 2‐nonanone there would be a methyl group on one side and a heptyl group on the other, with the presence of the heptyl chain causing steric difficulties.

### A revised scoring function for flexible as well as rigid guests

With this additional data, we were interested to see how well we could improve prediction of the binding strengths of guests using GOLD. The original scoring function (Eq. (1); see 7 for explanation of individual terms) was developed to predict binding of the original training set based largely on relatively rigid, cyclic guests.^7^ That training set of guests did contain four highly flexible guests which essentially did not bind and these have been excluded from the analysis in this paper.

(1) $$\log K{}_{\mathrm{calc}}=\;-4.48\times f\left(\mathrm{ligand}\;\;\mathrm{clash}\right)+0.20\times f\left(\mathrm{part}\;\;\mathrm{buried}\right)-0.10\times f(\mathrm{non}\text{-}\mathrm{polar})\;0.90\times f(\mathrm{ligand}\;\;\mathrm{torsion})-0.93\times f(\mathrm{ligand}\;\;\mathrm{flexibility})$$

**Figure 4:**
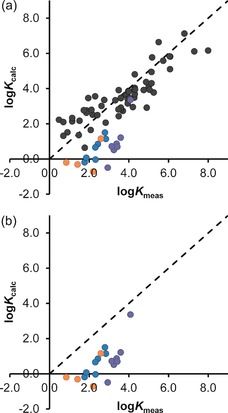
a) Calculated vs. measured binding constants for the original set of guests (black circles; see 7 for structures) plus new guests **1**–**18** (coloured circles), using the GOLD software with the scoring function in Eq. (1) (from 7). Error bars for the log*K*_meas_ values are similar to or smaller than the diameter of the circles used as data points. Blue circles are the C_7_ ketones (guests **1**–**7**); purple circles are the C_9_ ketones (guests **8**–**14**); orange circles are the alkyl phosphonates (guests **15**–**18**). b) The data for the new guests **1**–**18** only, to emphasise the persistent under‐estimation of binding strengths using the scoring function in Eq. (1). Addition of the new data points for **1**–**18** increases the RMSD value from 0.79 (black data points only) to 1.26 (all data points).

Figure 4 a shows the correlation of predicted and measured binding constants for the previously reported training set of guests (black points, with a few non‐bonding guests removed from the analysis),^7^ plus the new more flexible guests (coloured points; see Figure caption), using Equation (1) as the scoring function. In Figure 4 b is shown the same data for the new guests but with the data from the original training set not shown. It will be immediately apparent that there is a significant and systematic under‐estimation of the binding strengths of all of the new guests.

We ascribe this to the fact this initial scoring function (Eq. (1)) does not properly take account of both enthalpic and entropic effects associated with the loss of conformational flexibility when the guests bind, as the training set contained no highly flexible guests for which reliable binding data could be obtained. Notably, the data point closest to the *y=x* line (i.e., for which predicted binding most closely matches the measured value) is that for 1,1,1,3,3,3‐hexamethylacetone (**14**), with the highest degree of substitution and hence the lowest conformational flexibility.

To improve this situation we added the 18 new measured binding constants for new guests (Table 1) to the training set, and then re‐refined the scoring function: this allows the individual weightings of the different components to vary until the predicted and measured binding constants for the new expanded training set matched one another as closely as possible, as assessed by the RMSD value.(2)

(2) $$\log K{}_{\mathrm{calc}}=\;-4.48\times f\left(\mathrm{ligand}\;\;\mathrm{clash}\right)+0.22\times f\left(\mathrm{part}\;\;\mathrm{buried}\right)-0.10\times f(\mathrm{non}\;\;\mathrm{polar})+0.022\times f(\mathrm{ligand}\;\;\mathrm{torsion})-0.36\times f(\mathrm{ligand}\;\;\mathrm{flexibility})$$

The revised scoring function is shown in Equation (2). Comparison with Equation (1) is interesting as it shows which specific contributions have had their weightings changed to account for the flexibility of the additional guests. We can see, for example, that the ligand clash term, which quantifies unfavourable host/guest steric interactions, and the non polar term, which takes account of matching of hydrophobic surfaces, are completely unchanged. The part buried term, which takes account of the burial of a polar group in a non‐polar environment, has slightly (10 %) increased. However there are substantial relative changes to the weightings of the ligand torsion and ligand flexibility terms, which have both dramatically diminished. Significantly, these are the factors that take most account of changes in guest conformation on binding. The ligand torsion term accounts for the enthalpic penalty associated with conformational changes of flexible chains when a guest binds; the ligand flexibility term is, precisely, the NRB value from Table 1 which therefore takes account of the entropic cost of restricting the number of conformational possibilities for flexible guests. The fact that both of these contributions have substantially diminished suggests that the original scoring function in Equation (1) was over‐estimating the importance of these, which is why the calculated log*K*_calc_ values for the new flexible guests were consistently too low in Figure 4.

**Figure 5:**
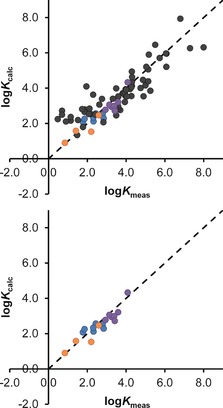
The same data as for Figure 4 except that calculated binding constants were obtained using the new scoring function in Eq. (2), which has been revised to take account of guest flexibility. Error bars for the log*K*_meas_ values are similar to or smaller than the diameter of the circles used as data points. The RMSD value is 0.77. The new guests **1**–**18** have their binding predicted very well and revision of the scoring function has not significantly affected binding predictions for the original guest set (black data points only) which had RMSD=0.79 (7).

Using the improved scoring function in Equation (2), the plot of calculated versus measured binding constants for the expanded training set is in Figure 5 a, using the same colouring convention as before with new guests being shown with coloured data points. Figure 5 b shows the same data for just the new guests with the original guests removed for clarity. It will be apparent that i) there is now a very good match between predicted and calculated binding for the new set of more flexible guests, as the over‐estimation of the enthalpic and entropic penalties associated with conformational restriction when flexible guests bind have been corrected (compare bottom panels of Figures 4 and 5); and ii) this has been achieved without compromising the prediction of binding constants for the original set of more rigid guests.

### Evaluation of different contributions to guest binding

Given the revised scoring function which predicts binding of these flexible guests very well, we can look at the magnitudes of the individual contributions to guest binding; these are the five parameters (with their associated weightings) in Equation (2) that are calculated by GOLD for each guest. These are summarised in Table 4 along with the associated log*K*_calc_ values. There are some interesting points to note here.

**Table 4:** Contributions to guest binding of the individual parameters caulcated using GOLD; the relative contributions of each component are given by the weighting coefficients form the new scoring functin in Eq. (2).

Guest	Ligand clash	Ligand torsion	Part buried	Non polar	Ligand flexibility^[a]^	Log *K*_calc_
	−4.48^[b]^	0.0219^[b]^	0.216^[b]^	−0.102^[b]^	0.360^[b]^	
**1**	0	0.085	−2.237	−38.99	4	2.07
**2**	0	0.179	−2.172	−40.61	4	2.25
**3**	0	0.218	−2.143	−39.15	4	2.11
**4**	0	0.063	−2.021	−39.84	3	2.56
**5**	0	0.113	−1.777	−37.00	3	2.33
**6**	0	0.186	−2.030	−35.97	2	2.53
**7**	0	0.048	−2.152	−33.84	2	2.28
**8**	0	0.291	−1.702	−51.64	6	2.77
**9**	0	0.587	−1.801	−49.95	5	2.94
**10**	0	0.688	−1.956	−51.42	5	3.06
**11**	0	1.043	−2.034	−48.12	5	2.71
**12**	0	0.279	−2.017	−47.40	4	2.98
**13**	0	0.419	−2.128	−49.78	4	3.21
**14**	0	0.335	0.259	−48.65	2	4.33
**15**	0	0.052	−3.573	−23.29	2	0.89
**16**	0	0.489	−3.251	−36.19	4	1.58
**17**	0	0.733	−3.519	−39.74	5	1.53
**18**	0	1.160	−3.777	−45.84	4	2.46

[a] This is the NRB parameter form Table 1. [b] Weighting coefficient for each parameter from the scoring function (see Eq. (2)).

Firstly, in all cases, the ligand clash parameter is zero; this is a measure of unfavourable host/guest steric interactions. As all of the guests used have a molecular volume that is considerably below the Rebek 55 % limit,6d, 11 it is reasonable that these should be zero as there are no steric clashes: if there were, the guest would not bind in the first place. In agreement with this the high weighting of this parameter would result in a large negative contribution to log*K*_calc_ so these guests would not be under consideration.

Secondly, by far the two largest remaining contributions to the log*K*_calc_ values (parameter value * weighting) are the terms non polar (matching of hydrophobic surfaces, which is favourable) and ligand flexibility (loss conformational entropy, which is unfavourable). If we consider guest **8** (5‐nonanone) the data in Table 4 show a favourable contribution to log*K*_calc_ of 5.29 arising from matching of hydrophobic surfaces, and an unfavourable contribution of 2.16 arising from loss of conformational entropy. Compared to these, the other contributions (part buried and ligand torsion) are one and three orders of magnitude less significant. For the most strongly binding guest **14** we find a similar result. The hydrophobic contribution to log*K*_calc_ is similar (+4.98) to that of **8** due to its similar molecular surface area, and this clearly dominates log*K*_calc_. The unfavourable contribution to log*K*_calc_ arising from loss of conformational entropy is now smaller (0.72) as expected from the smaller NRB value of this less flexible guest, but this still very substantially outweighs the part buried (0.06) and ligand torsion (<0.01) contributions. Notably, the ligand torsion term, which can be considered as the enthalpic cost associated of restricting guest conformation, is completely insignificant compared to the ligand flexibility (entropy) term.

Overall therefore, for this series of guests, the parameters contributing to binding derived from GOLD are principally i) a strongly favourable hydrophobic effect that scales with guest surface area, and ii) an unfavourable entropy effect arising from loss of conformational flexibility that scales with the number of rotatable bonds and explains why large open‐chain guests show weak or no binding. Other parameters, as included in the scoring function, carry much less weight than those two; this is consistent with observations of the principal factors responsible for substrate binding to proteins in many experiments and calculations.12g

## Conclusion

Using two sets of isomeric guests based on C_7_ and C_9_ ketones, as well as some simple alkyl phosphonates, we have investigated the effects of guest flexibility on their ability to bind in the cavity of a coordination cage host. In addition to expected differences between the C_7_ and C_9_ guest sets arising from the greater hydrophobic surface area of the latter, within each isomeric guest series there are clear differences in binding strength associated with changes in ligand flexibility, as expressed by the parameter NRB (number of rotatable bonds). Thus guests with a high NRB value–which denotes linear, unsubstituted alkyl chains–bind consistently more weakly than isomeric guests with a low NRB value, which denotes highly branched alkyl chains with less conformational flexibility. An approximately consistent increment of 2 kJ mol^−1^ for binding free energy was observed in each series of isomeric guests as the NRB parameter decreased by 1 due to a higher degree of alkyl chain substitution; this is principally entropic in origin and agrees with previous data on binding of flexible ligands to protein hosts. With this new binding constant data for 18 flexible guests, we were able to improve the scoring function used by the molecular docking programme GOLD so that it predicts accurately binding constants for both rigid and flexible guests, extending the utility of our in silico screening method for identifying potential new guests for the cage host.

## Experimental Section

### General details

The cages **H** (14) and **H^w^** (9a) were prepared as previously described. The guests **1**–**18** were obtained from Sigma–Aldrich and used as received. The binding constants in Table 1 were measured by standard ^1^H NMR spectroscopic titrations (D_2_O, 298 K) on a Bruker AV‐III 400 MHz instrument as previously reported;5e, 6c, 7, 9 illustrative examples are shown in the Supporting Information. Calculations with GOLD and development of the new scoring function followed previously reported methodology.^7^ Molecular volumes and surface areas in Table 1 were calculated from the 0.002 Bohr Å^−3^ isodensity surface from B3LYP 6‐31G* DFT calculations implemented in Spartan′06.^15^

### X‐ray crystallography

Crystals of cage/guest complexes were prepared from crystals of **H** (grown from MeOH) which were treated with a concentrated methanolic solution of the appropriate guest, which resulted in uptake of the guest into the cage cavities in the crystals. Data for **H⋅6⋅MeOH** were collected on a Bruker Apex‐II diffractometer using Mo‐Kα radiation; data for **H⋅3⋅MeOH** were collected on a Bruker D8 Venture diffractometer using Cu‐Kα radiation. In each case a crystal was removed from the mother liquor, coated with oil, and transferred rapidly to a stream of cold N_2_ on the diffractometer to prevent any decomposition due to solvent loss. In all cases, after integration of the raw data, and before merging, an empirical absorption correction was applied (SADABS)^16^ based on comparison of multiple symmetry‐equivalent measurements. The structures were solved by direct methods and refined by full‐matrix least squares on weighted *F* ^2^ values for all reflections using the SHELX suite of programs.^17^ Pertinent crystallographic data are collected in Table 2. CCDC 1509475 and 1509476 contain the supplementary crystallographic data for this paper. These data are provided free of charge by The Cambridge Crystallographic Data Centre.

In both cases crystals exhibited the usual problems of this type of structure, in particular weak scattering due to a combination of poor crystallinity, solvation, and disorder of anions / solvent molecules. Both structures contained large solvent‐accessible voids containing diffuse electron density which could not meaningfully be modelled, ascribed to severely disordered solvent molecules as well as those tetrafluoroborate anions that could not be located. This diffuse electron density was removed from the refinements using the SQUEEZE function in PLATON.^18^ To assist in the refinements, the number of parameters was kept as low as possible by extensive use of geometric restraints on aromatic rings and anions (e.g. pyridyl and pyrazolyl rings were refined as idealised hexagons and pentagons, respectively; and tetrafluoroborate anions as idealised tetrahedra), as well as global restraints on atomic displacement parameters. Full details are in the individual CIFs.

In both cases the cage cavities contain a 50:50 disordered combination of one ketone guest molecule (**3** or **6**) in one binding pocket, and one methanol molecule in the opposite binding pocket. Thus the asymmetric unit contains half of a complete cage astride an inversion centre, containing in its binding pocket a superimposed combination of 50 % of the guest ketone and 50 % of a MeOH molecule. Significant metric parameters associated with bound guests are the main text; bond distances and angles associated with the host cage are unremarkable and not tabulated.

## Supporting information

As a service to our authors and readers, this journal provides supporting information supplied by the authors. Such materials are peer reviewed and may be re‐organized for online delivery, but are not copy‐edited or typeset. Technical support issues arising from supporting information (other than missing files) should be addressed to the authors.

Supplementary

## References

[1] Recent reviews:

[1a] M. Yoshizawa , J. K. Klosterman , M. Fujita , Angew. Chem. Int. Ed. 2009, 48, 3418; 10.1002/anie.200805340 19391140

[1b] T. R. Cook , Y.-R. Zheng , P. J. Stang , Chem. Rev. 2013, 113, 734; 23121121 10.1021/cr3002824 PMC3764682

[1c] M. D. Pluth , R. G. Bergman , K. N. Raymond , Acc. Chem. Res. 2009, 42, 1650; 19591461 10.1021/ar900118t

[1d] M. M. J. Smulders , I. A. Riddell , C. Browne , J. R. Nitschke , Chem. Soc. Rev. 2013, 42, 1728; 23032789 10.1039/c2cs35254k

[1e] S. Zarra , D. M. Wood , D. A. Roberts , J. R. Nitschke , Chem. Soc. Rev. 2015, 44, 419; 25029235 10.1039/c4cs00165f

[1f] R. J. Chakrabarty , P. S. Mukherjee , P. J. Stang , Chem. Rev. 2011, 111, 6810; 21863792 10.1021/cr200077m PMC3212633

[1g] H. Vardhan , M. Yusubov , F. Verpoort , Coord. Chem. Rev. 2016, 306, 171;

[1h] A. Schmidt , A. Casini , F. E. Kuhn , Coord. Chem. Rev. 2014, 275, 19;

[1i] R. Custelcean , Chem. Soc. Rev. 2014, 43, 1813; 24384869 10.1039/c3cs60371g

[1j] M. Han , D. M. Engelhard , G. H. Clever , Chem. Soc. Rev. 2014, 43, 1848; 24504200 10.1039/c3cs60473j

[1k] H. Amouri , C. Desmarets , J. Moussa , Chem. Rev. 2014, 114, 2015. 10.1021/cr200345v 22251425

[2a] A. Galan , P. Ballester , Chem. Soc. Rev. 2016, 45, 1720; 26797259 10.1039/c5cs00861a

[2b] H. Vardhan , F. Verpoort , Adv. Synth. Catal. 2015, 357, 1351.

[3a] C. G. P. Taylor , J. R. Piper , M. D. Ward , Chem. Commun. 2016, 52, 6225; 10.1039/c6cc02021f 27020844

[3b] J. Wang , C. He , P. Wu , J. Wang , C. Duan , J. Am. Chem. Soc. 2011, 133, 12402; 21749065 10.1021/ja2048489

[3c] C. He , Z. Lin , Z. Je , C. Duan , C. Xu , Z. Wang , C. Yan , Angew. Chem. Int. Ed. 2008, 47, 2463; 10.1002/anie.200704206 18076007

[3d] S. Shanmugaraju , P. S. Mukherjee , Chem. Eur. J. 2015, 21, 6656; 25694365 10.1002/chem.201406092

[3e] P. P. Neelakandan , A. Jimenez , J. R. Nitschke , Chem. Sci. 2014, 5, 908.

[4a] C. J. Brown , F. D. Toste , R. G. Bergman , K. N. Raymond , Chem. Rev. 2015, 115, 3012; 25898212 10.1021/cr4001226

[4b] W. Cullen , M. C. Misuraca , C. A. Hunter , N. H. Williams , M. D. Ward , Nat. Chem. 2016, 8, 231; 26892554 10.1038/nchem.2452

[4c] L. Catti , Q. Zhang , K. Tiefenbacher , Chem. Eur. J. 2016, 22, 9060; 27150251 10.1002/chem.201600726

[4d] C. Deraedt , D. Astruc , Coord. Chem. Rev. 2016, 324, 106.

[5a] J. W. Yi , N. P. E. Barry , M. A. Furrer , O. Zava , P. J. Dyson , B. Therrien , B. H. Kim , Bioconjugate Chem. 2012, 23, 461; 10.1021/bc200472n 22263930

[5b] B. Therrien , Chem. Eur. J. 2013, 19, 8378; 23737435 10.1002/chem.201301348

[5c] J. E. M. Lewis , E. L. Gavey , S. A. Cameron , J. D. Crowley , Chem. Sci. 2012, 3, 778;

[5d] A. Barci , J.-P. Mbakidi , V. Chaleix , V. Sol , E. Orhan , B. Therrien , Organometallics 2015, 34, 4138;

[5e] W. Cullen , S. Turega , C. A. Hunter , M. D. Ward , Chem. Sci. 2015, 6, 625; 28936311 10.1039/c4sc02090a PMC5588781

[5f] K. Ariga , M. Naito , Q. Ji , D. Payra , CrystEngComm 2016, 18, 4890;

[5g] B. Therrien , Top. Curr. Chem. 2011, 319, 35. 10.1007/128_2011_272 21952848

[6a] M. M. J. Smulders , S. Zarra , J. R. Nitschke , J. Am. Chem. Soc. 2013, 135, 7039; 23544861 10.1021/ja402084x

[6b] J. L. Brumaghim , M. Michels , K. N. Raymond , Eur. J. Org. Chem. 2004, 22, 4552;

[6c] S. Turega , W. Cullen , M. Whitehead , C. A. Hunter , M. D. Ward , J. Am. Chem. Soc. 2014, 136, 8475; 24841689 10.1021/ja504269m

[6d] S. Mecozzi , J. Rebek , Chem. Eur. J. 1998, 4, 1016.

[7] W. Cullen , S. Turega , C. A. Hunter , M. D. Ward , Chem. Sci. 2015, 6, 2790. 28706666 10.1039/c5sc00534e PMC5489031

[8a] G. Jones , P. Willett , R. C. Glen , J. Mol. Biol. 1995, 245, 43; 7823319 10.1016/s0022-2836(95)80037-9

[8b] G. Jones , P. Willett , R. C. Glen , A. R. Leach , R. Taylor , J. Mol. Biol. 1997, 267, 727; 9126849 10.1006/jmbi.1996.0897

[8c] J. W. M. Nissink , C. Murray , M. Hartshorn , M. L. Verdonk , J. C. Cole , R. Taylor , Proteins 2002, 49, 457; 12402356 10.1002/prot.10232

[8d] M. L. Verdonk , J. C. Cole , M. J. Hartshorn , C. W. Murray , R. D. Taylor , Proteins Struct. Funct. Bioinf. 2003, 52, 609 *Zeitschrift wurde erst 2004 geründet*! ;

[8e] J. C. Cole , J. W. M. Nissink , R. Taylor , in Virtual Screening in Drug Discovery (Eds. J. Alvarez, B. Shoichet), CRC Press, Boca Raton, Florida, USA, 2005;

[8f] M. L. Verdonk , G. Chessari , J. C. Cole , M. J. Hartshorn , C. W. Murray , J. W. M. Nissink , R. D. Taylor , R. Taylor , J. Med. Chem. 2005, 48, 6504; 16190776 10.1021/jm050543p

[8g] GOLD software: http://www.ccdc.cam.ac.uk/solutions/csd-discovery/components/gold/.

[9a] M. Whitehead , S. Turega , A. Stephenson , C. A. Hunter , M. D. Ward , Chem. Sci. 2013, 4, 2744;

[9b] W. Cullen , K. A. Thomas , C. A. Hunter , M. D. Ward , Chem. Sci. 2015, 6, 4025. 28717464 10.1039/c5sc01475a PMC5497272

[10a] S. Turega , M. Whitehead , B. R. Hall , A. J. H. M. Meijer , C. A. Hunter , M. D. Ward , Inorg. Chem. 2013, 52, 1122; 23301770 10.1021/ic302498t

[10b] A. J. Metherell , M. D. Ward , Dalton Trans. 2016, 45, 16096. 27604482 10.1039/c6dt03041f

[11a] M. R. Ams , D. Ajami , S. L. Craig , J. S. Yang , J. Rebek , J. Am. Chem. Soc. 2009, 131, 13190; 19754179 10.1021/ja903198v

[11b] J. Rebek , Acc. Chem. Res. 2009, 42, 1660. 19603810 10.1021/ar9001203

[12a] D. F. Veber , S. R. Johnson , H.-Y. Cheng , B. R. Smith , K. W. Ward , K. D. Kopple , J. Med. Chem. 2002, 45, 2615; 12036371 10.1021/jm020017n

[12b] H.-J. Böhm , J. Computer-Aided Mol. Des. 1994, 8, 243; 10.1007/BF00126743 7964925

[12c] M. D. Eldridge , C. W. Murray , T. R. Auton , G. V. Paolini , R. P. Mee , J. Computer-Aided Mol. Des. 1997, 11, 425; 10.1023/a:1007996124545 9385547

[12d] C. A. Chang , W. Chen , M. K. Gilson , Proc. Natl. Acad. Sci. USA 2007, 104, 1534; 17242351 10.1073/pnas.0610494104 PMC1780070

[12e] D. L. Mobley , K. A. Dill , Structure 2009, 17, 489; 19368882 10.1016/j.str.2009.02.010 PMC2756098

[12f] S. Vajda , Z. Weng , R. Rosenfeld , C. DeLisi , Biochemistry 1994, 33, 13977; 7947806 10.1021/bi00251a004

[12g] M. K. Gilson , H.-X. Zhou , Ann. Rev. Biophys. Biomol. Struct. 2007, 36, 21. 17201676 10.1146/annurev.biophys.36.040306.132550

[13a] N. T. Southall , K. A. Dill , A. D. J. Haymet , J. Phys. Chem. B 2002, 106, 521;

[13b] G. Hummer , S. Garde , A. E. García , M. E. Paulaitis , L. R. Pratt , J. Phys. Chem. B 1998, 102, 10469;

[13c] L. R. Pratt , A. Pohorille , Chem. Rev. 2002, 102, 2671; 12175264 10.1021/cr000692+

[13d] C. Tanford , Science 1978, 200, 1012; 653353 10.1126/science.653353

[13e] K. N. Houk , A. G. Leach , S. P. Kim , X. Zhang , Angew. Chem. Int. Ed. 2003, 42, 4872; 10.1002/anie.200200565 14579432

[13f] E. A. Meyer , R. K. Castellano , F. Diederich , Angew. Chem. Int. Ed. 2003, 42, 1210; 10.1002/anie.200390319 12645054

[14] I. S. Tidmarsh , T. B. Faust , H. Adams , L. P. Harding , L. Russo , W. Clegg , M. D. Ward , J. Am. Chem. Soc. 2008, 130, 15167. 18855358 10.1021/ja805605y

[15] Spartan′06; Wavefunction, Inc., Irvine, CA; **2006**.

[16] G. M. Sheldrick, SADABS: A program for absorption correction with the Siemens SMART system, University of Göttingen, Germany, **2008**.

[17] G. M. Sheldrick , Acta Crystallogr. Sect. A 2008, 64, 112.18156677

[18a] A. Spek , J. Appl. Crystallogr. 2003, 36, 7;

[18b] P. van der Sluis , A. L. Spek , Acta Crystallogr. Sect. A 1990, 46, 194.

