# Supplementary material for: A Quantitative Study of the Effects of Guest Flexibility on Binding Inside a Coordination Cage Host
Source: Chemistry. 2016 Nov 23;23(1):206–13. doi: 10.1002/chem.201604796 (PMC6680264; doi:10.1002/chem.201604796)
Supplement: Supplementary file 1 — Supplementary [file CHEM-23-206-s001.pdf]

CHEMISTRY
A European Journal

Supporting Information

A Quantitative Study of the Effects of Guest Flexibility on Binding Inside a Coordination Cage Host

Christopher G. P. Taylor+, William Cullen+, Olivia M. Collier, and Michael D. Ward*[a]

chem_201604796_sm_miscellaneous_information.pdf

Measurements of binding constants

Binding constants of guests were measured by NMR spectroscopy in D_2O at 298K using a Bruker AV3-400 spectrometer. Concentration of host cage **H** was always 0.2 mM. Guest binding could be in either fast or slow exchange depending on guest size; illustrations of each type of behaviour, with data analysis, are shown below.

1. Example Guest in Slow Exchange

For guests in slow exchange, separate signals for free host (**H**) and host/guest complex (**HG**) could be seen during a titration, as shown below.

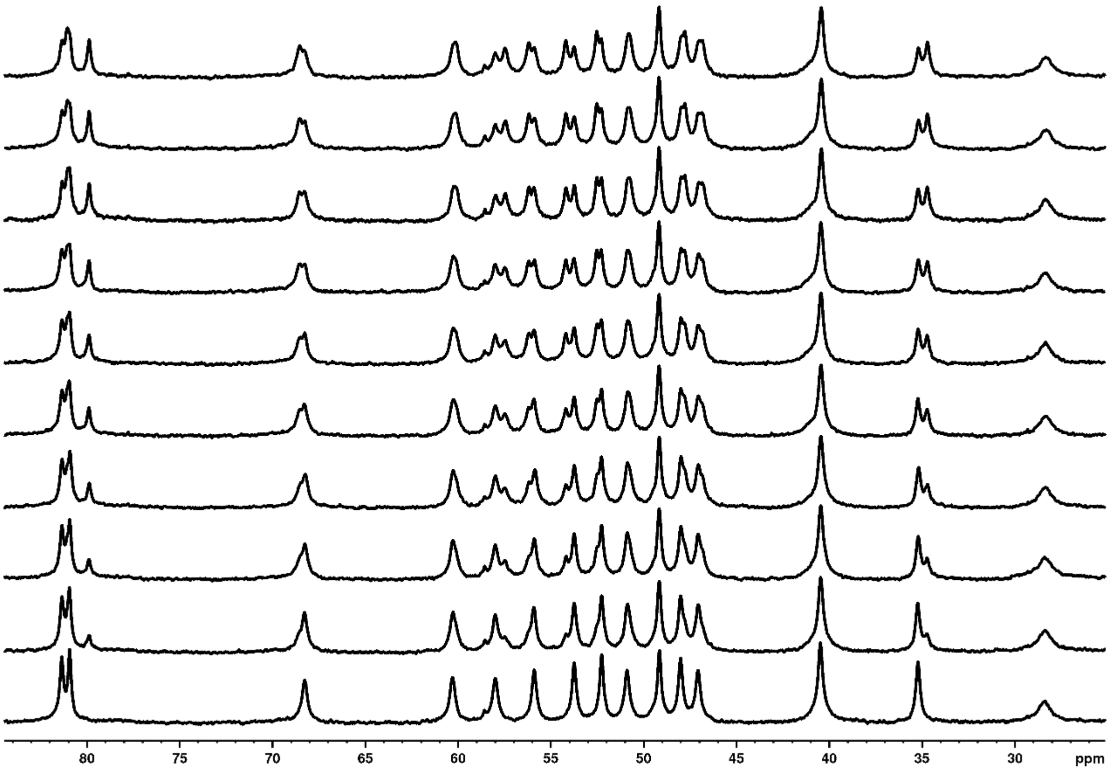

Figure S1: Section of NMR peaks in slow exchange for the titration of **H** with 4,4-dimethyl-2-pentanone. The fitted data based on peak integrations are shown in Table S1 below.

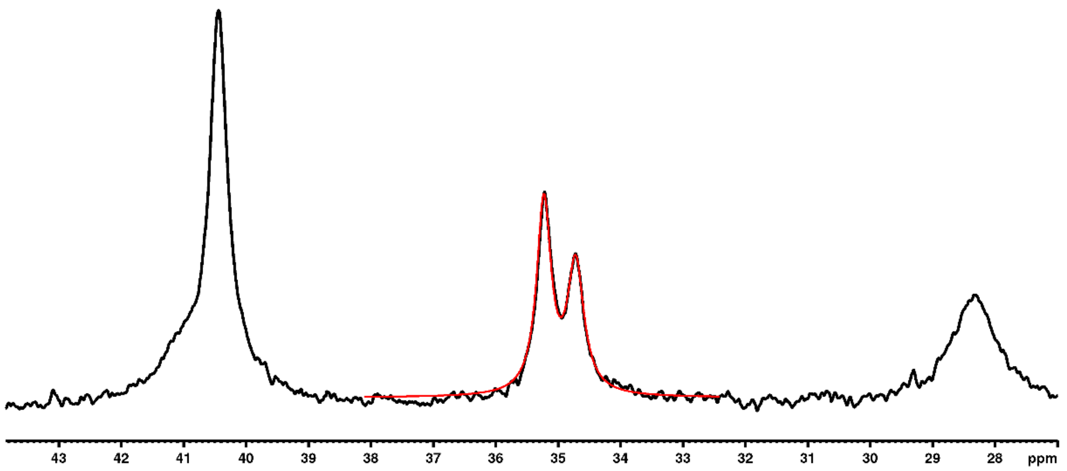

Figure S2: Example of the fitted curve from deconvolution of the overlapping signals for **H** and **HG**, used to give the [H]/[HG] ratio (integral ratio 4.582 vs. 4.037, giving ratio of 1.1 : 1, see line 5 in **Table S1**).

[H]/[HG]	[G]o	[H]	[HG]	[G]	K_a	ΔG
2.2	7.50E-04	1.38E-04	6.24E-05	6.88E-04	6.59E+02	-16.1
1.7	1.00E-03	1.25E-04	7.45E-05	9.25E-04	6.42E+02	-16.0
1.4	1.25E-03	1.15E-04	8.50E-05	1.17E-03	6.34E+02	-16.0
1.3	1.50E-03	1.15E-04	8.55E-05	1.41E-03	5.28E+02	-15.5
1.1	1.75E-03	1.06E-04	9.37E-05	1.66E-03	5.32E+02	-15.6
0.7	2.25E-03	8.54E-05	1.15E-04	2.14E-03	6.29E+02	-16.0
0.8	2.50E-03	8.64E-05	1.14E-04	2.39E-03	5.51E+02	-15.6
2.3	7.50E-04	1.39E-04	6.14E-05	6.89E-04	6.43E+02	-16.0
1.2	1.50E-03	1.11E-04	8.92E-05	1.41E-03	5.71E+02	-15.7
0.7	2.50E-03	8.50E-05	1.15E-04	2.39E-03	5.67E+02	-15.7
1.1	2.00E-03	1.04E-04	9.62E-05	1.90E-03	4.87E+02	-15.3
0.7	2.00E-03	8.55E-05	1.15E-04	1.89E-03	7.11E+02	-16.3

Table S1: The **[H]/[HG] r**atios for a range of starting guest concentrations, determined by integration of signals in the slow exchange NMR spectra. The free guest, host and host-guest complex concentrations can then be determined and converted to obtain the equilibrium constant and Gibb's free energy. Several repeats were averaged to give the final binding constant quoted in the main text.

2. Example Guest in Fast Exchange

Changes in the ^{1}H NMR spectra recorded during titration of the host cage (0.2 mM) with 5-methyl-2-hexanone (0 to 10 mM) in D_2O are shown as an example. As this guest is in fast exchange between free / bound states, we see a steady shift in some signals for the host cage, as shown in Fig. S4; these curves could be fitted to a 1:1 binding isotherm, and the quoted binding constant is taken from the average of the individual curve fits from several repeat titration experiments.

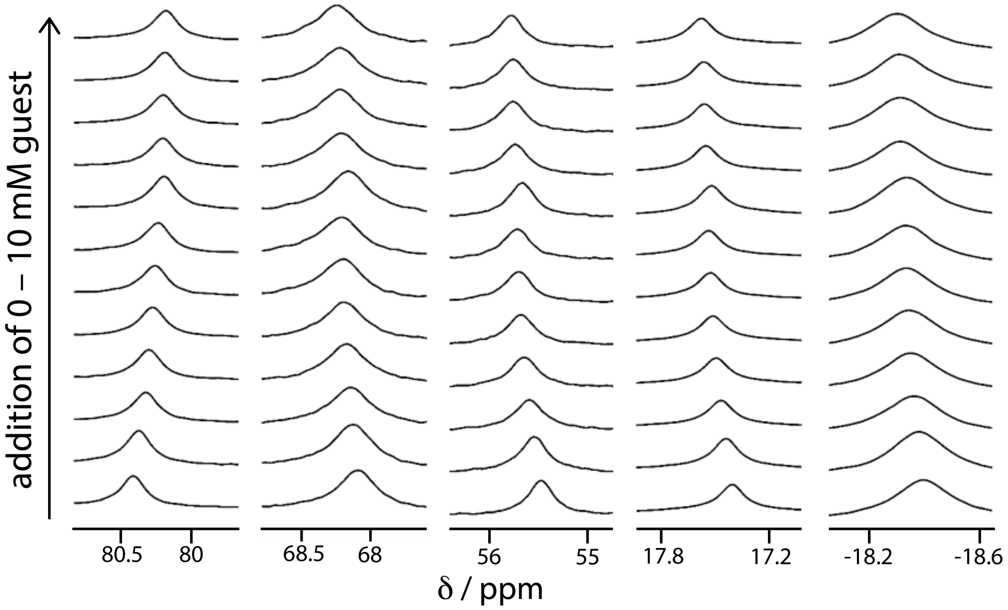

Figure S3: Variation in some NMR signals of **H** during titration with 5-methyl-2-hexanone.

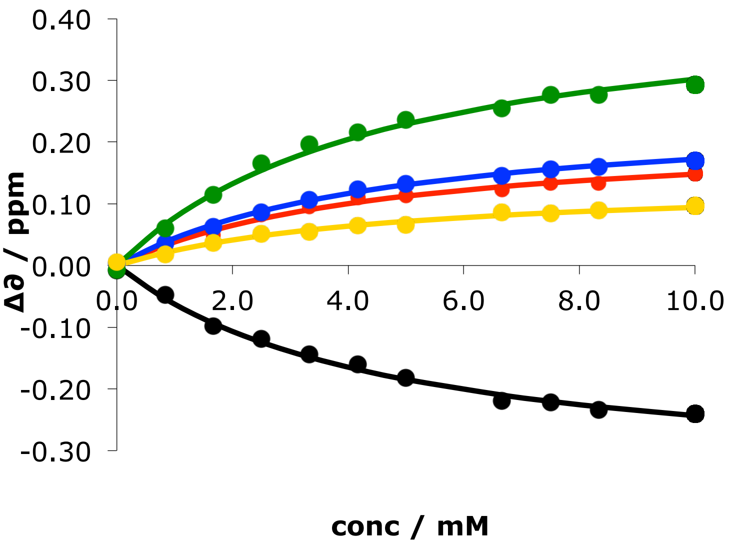

Figure S4: Plot showing the steady shifts in ^{1}H NMR signals based on the data in Fig. S4. All titrations were repeated three times, and binding constants calculated based on shifts of several individual signals in each titration.
